# miRNASNP: a database of miRNA related SNPs and their effects on miRNA function

**DOI:** 10.1186/1471-2105-13-S18-A2

**Published:** 2012-12-14

**Authors:** Jing Gong, Yin Tong, Hong-Mei Zhang, An-Yuan Guo

**Affiliations:** 1 Hubei Bioinformatics & Molecular Imaging Key Laboratory, Department of Biomedical Engineering, College of Life Science and Technology, Huazhong University of Science and Technology, Wuhan, 430074, China

## Background

MicroRNAs (miRNAs) are a family of endogenous small non-coding RNAs involved in various developmental and physiological processes by negatively regulating gene expression. Single nucleotide polymorphisms (SNPs) are important variations for the diversity among individuals, as well as leading to phenotypes, traits, and diseases. What will happen when SNPs meet miRNAs? To date, a number of studies have demonstrated that SNPs in target sites or miRNA genes were associated with diseases [1-3]. Our aim is to characterize these functional miRNA related SNPs comprehensively.

## Results

Through mapping SNPs onto miRNAs, we totally identified 757 SNPs (including indel polymorphisms) in 440 human pre-miRNAs, 218 SNPs in pre-miRNAs of other 8 species and thousands of SNPs in pre-miRNAs flanking regions. Of them, 50 SNPs are in the seed regions of 41 human miRNAs. Two different methods (TargetScan and miRanda) were used to predict the target sites for the wild type miRNAs and SNP-miRNAs. By comparing the targets of wild type miRNA with that of SNP-miRNA, we obtained the potential miRNA targets loss and gain. We found about half of the targets will be changed by SNP in miRNA seed region. In addition, we experimentally confirmed seven loss of-function SNPs and one gain-of-function SNP by luciferase report assay.

We also mapped SNPs onto gene 3’ untranslated region (3’UTR) and identified tens of thousands of SNPs in 3’UTRs which would affect the miRNA/gene interaction, either destroy a real target or create an illegal target. Among these miRNA related SNPs, we think the SNPs with relatively high minor allele frequencies (MAF), high MAF difference between populations and undergoing positive selection pressure will be important candidates for population phonotype research and complex trait studies.

All useful data were compiled into miRNASNP, a user-friendly free online database (http://www.bioguo.org/miRNASNP/).

## Conclusions

We have systematically identified and analyzed the human polymorphisms in miRNAs and miRNA target sites and analyzed their potential influences on target binding. Our database will be a useful resource for studying miRNA function, identifying disease-associated miRNAs, and further personalized medicine.
